# Carbon quantum dots in bioimaging and biomedicines

**DOI:** 10.3389/fbioe.2023.1333752

**Published:** 2024-01-22

**Authors:** Surya Das, Somnath Mondal, Dhiman Ghosh

**Affiliations:** ^1^ Department of Chemistry, University of Kalyani, Kalyani, India; ^2^ Department of Chemistry, Pennsylvania State University, State College, PA, United States; ^3^ Department of Chemistry and Applied Biosciences, Zurich, Switzerland

**Keywords:** carbon quantum dots, biocompatible quantum dots, bioimaging, biomedicine, metal-doped CQD, CQD-polymer composite (CPD), graphene-based carbon dots (GQD), carbon dots

## Abstract

Carbon quantum dots (CQDs) are gaining a lot more attention than traditional semiconductor quantum dots owing to their intrinsic fluorescence property, chemical inertness, biocompatibility, non-toxicity, and simple and inexpensive synthetic route of preparation. These properties allow CQDs to be utilized for a broad range of applications in various fields of scientific research including biomedical sciences, particularly in bioimaging and biomedicines. CQDs are a promising choice for advanced nanomaterials research for bioimaging and biomedicines owing to their unique chemical, physical, and optical properties. CQDs doped with hetero atom, or polymer composite materials are extremely advantageous for biochemical, biological, and biomedical applications since they are easy to prepare, biocompatible, and have beneficial properties. This type of CQD is highly useful in phototherapy, gene therapy, medication delivery, and bioimaging. This review explores the applications of CQDs in bioimaging and biomedicine, highlighting recent advancements and future possibilities to increase interest in their numerous advantages for therapeutic applications.

## 1 Introduction

An element’s properties are determined by its electronic configuration, but Quantum phenomena emerge when matter is reduced to nanoscale dimensions due to matter size (Cotta, 2020; García de Arquer et al., 2023). The Chemistry Nobel Prize for 2023 recognizes the discovery and evolution of quantum dots (QDs) and is jointly awarded to Aleksey Yekimov (created size-dependent quantum effects in coloured glass), Louis Brus (first to demonstrate size-dependent particle quantum effects), Moungi Bawendi (revolutionized the chemical productions of QD) (NobelPrize. org., 2024). QDs are tiny nanoparticles with properties determined by size. Quantum dots, the nanotechnology components offer significant benefits to humanity, including tiny sensors, flexible electronics, encrypted quantum communication, thinner solar cells, and surgery to guide tissue removal (Gangasani and Student, 2007; Cheki et al., 2013; Knoblauch et al., 2014; Zhao and Zhu, 2016).

Carbon nanomaterials have drawn great interest due to their simple surface functionalization, easy synthesis methods, biodegradability, and no toxicity. Carbon-based quantum dots or Carbon quantum dots (CQDs) are submicron-sized nanomaterials with zero dimensions that were initially produced in 2004 while purifying single-walled carbon nanotubes (SWCNTs) (Xu et al., 2004). Following this, CQDs have been applied to a broad range of applications because of their inexpensive preparation cost and advantageous qualities, which include solubility in aqueous medium, chemical inertness, biocompatibility, and non-toxicity (Wang et al., 2014; Lim et al., 2015; Sousa et al., 2021). CQDs exhibit unique and fascinating properties, with their colours varying based on their size (Mai et al., 2020). CQDs are largely employed in biological applications due to their intrinsic fluorescence, stability, biocompatibility, ease of manufacture, and lack of adverse effects (Lan et al., 2017; Pourmadadi et al., 2023). Carbon nanotubes, graphene, and fullerenes have low aqueous solubility resulting in no fluorescence emission in visible regions and having limited applications (Sahu et al., 2012; Liang et al., 2013; Yang et al., 2013). On the other hand, CQDs are not having such limitations. CQDs, with their unique optical and electronic properties, are increasingly used in electronics, sensors, solar cells, catalysis, supercapacitors, and energy conversion gadgets (Wang and Hu, 2014; Lim et al., 2015; Ahmad et al., 2017; Pal et al., 2019). Most CQD molecules are typically composed of a sp^2^/sp^3^ hybridized carbon core with various surface functional groups (Bhunia et al., 2013; Zhao et al., 2015). The water-soluble nature of CQDs is attributed to the presence of functional groups that contain oxygen, namely, carboxyl (-COOH) and hydroxyl (-OH), on their surface (Zhu et al., 2015). In addition to providing fluorescence characteristics, these functional groups on CQDs assist their solubility and enable them to generate durable colloids in water or polar solvents. Their significant quantum yield (QY) of fluorescence and stable red and near-infrared emission properties make them used in various applications in biology and medicine, particularly in bioimaging and biomedicines (Bhunia et al., 2013; Luo et al., 2013; Zuo et al., 2015). CQDs have also been employed to understand the interactions with various proteins, like other biocompatible nanomaterials (Mondal et al., 2015; 2016; Mondal, 2017; Malik et al., 2019). This opens a new avenue for understanding the significance of CQD in nanomedicines with possible interactions and their outcomes with various human proteins. CQDs’ distinct chemistry and optical and physical characteristics render them a potential candidate for advanced nanomaterials research for bioimaging and biomedicines (Nazir et al., 2014; Sasaki et al., 2021; Sangjan et al., 2022; Gedda et al., 2023).

CQDs and polymer composite materials are efficient for biological, biochemical, and biomedical applications due to their ease of preparation, biocompatibility, and economical (Kausar, 2019; Molaei, 2019; Feng et al., 2021; Wang et al., 2022; Pathak et al., 2023). To create photoluminescent materials, careful tuning of their surface-level chemical groups and size is necessary for proper electrical structure tuning (Pal et al., 2019; Abbas et al., 2023; Yang et al., 2023). CQDs can be synthesized from various sources and mixed with polymers to create composites. Developing better CQD/polymer composite materials utilizing ligand exchange, grafting, and capping, leads to commend biocompatibility and optoelectrical features is a growing research topic (Qi and Gao, 2008; Kausar, 2019; Pathak et al., 2023). A variety of *in vivo* biological obstacles, such as glomerular filtration barriers, blood-brain barriers, and ion channels, can be overcome by them due to their small size (Yang et al., 2009). This type of CQD has great applications in bioimaging, phototherapy, drug delivery, and gene therapy (Ross et al., 2020; Pathak et al., 2023; Soumya et al., 2023).

We present a concise overview of the applications of CQDs in bioimaging and biomedicine. This review will explore recent advances, future possibilities, and various applications of CQDs and CQDs-based composites in the field of bioimaging and biomedicines, aiming to increase interest in numerous advantages of CQDs in these fields.

## 2 Synthesis of CQDs

**FIGURE 1:**
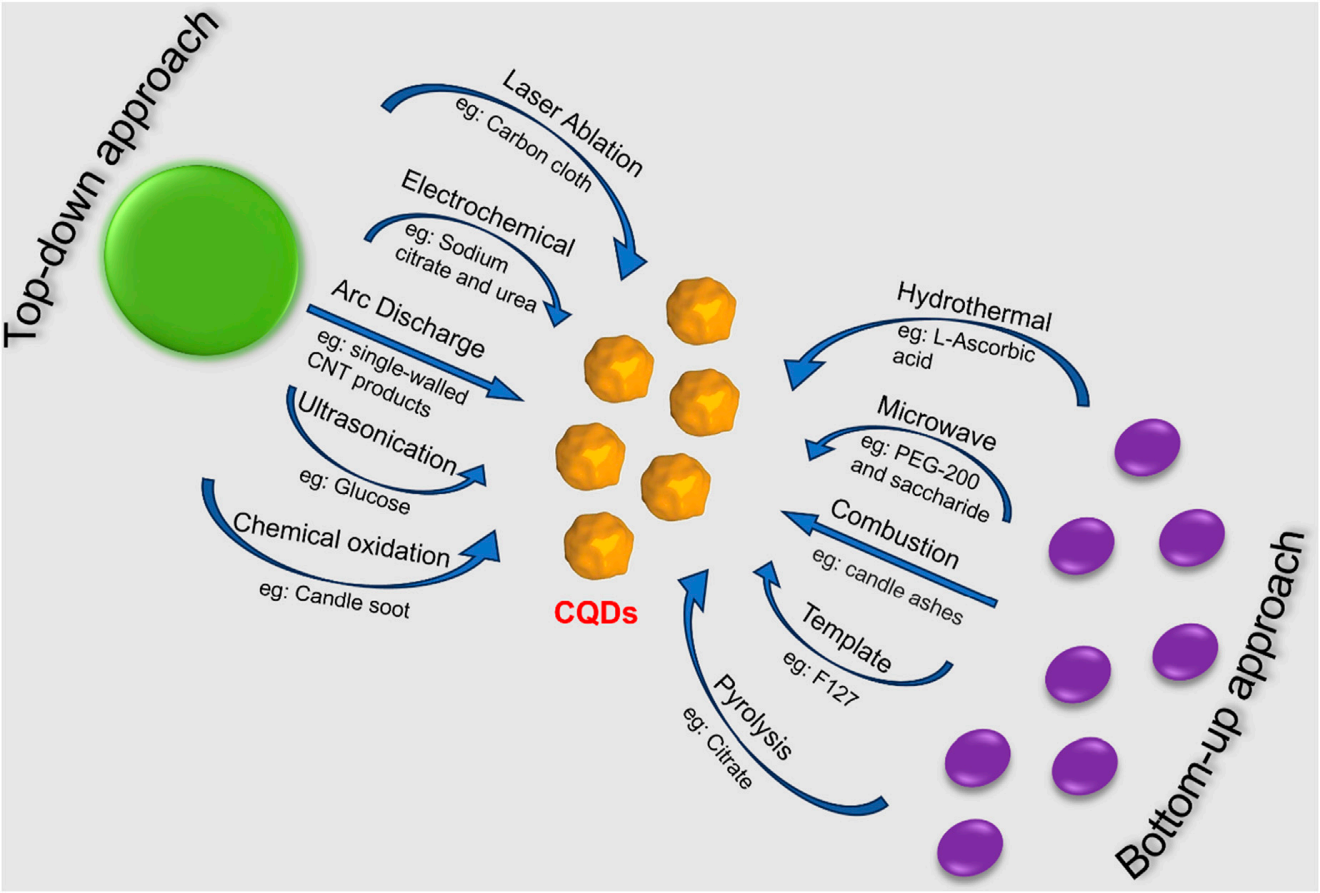
Top-down and bottom-up methods for CQD synthesis via various routes.

Through the purification method of single-walled carbon nanotubes, Scrivens et al. obtained luminous carbon nanoparticles for the first time in 2004. Since then, several synthetic routes have been reported for the synthesis of CQDs varying in size and surface functional groupings. In general, CQDs can be synthesized using either of two primary methods: top-down or bottom-up (Figure 1) (Niu N. et al., 2017; Yadav et al., 2023). Top-down approaches use physical or chemical mechanisms to split up bigger carbon structures into smaller ones. For instance, arc discharge, laser ablation, and electrochemical oxidation can be utilised to cut graphite or carbon nanotubes into CQDs. These techniques have the potential to yield high-quality CQDs with uniform size distribution and good crystallinity, but they also come with a cost and could produce hazardous byproducts. Conversely, using thermal or chemical reactions, CQDs are assembled from smaller carbon precursors in bottom-up approaches. To create CQDs from organic molecules or biomass, for instance, hydrothermal treatment, solvothermal synthesis, and microwave pyrolysis can be applied (Ren R. et al., 2019). These techniques can provide CQDs with different functional groups and doping components, however, they could also lead to poor size control and low yield. To enhance the characteristics and functionality of CQDs, post-treatment procedures such as doping, surface passivation, and purification can be applied to both top-down and bottom-up approaches. The intended uses and properties of CQDs influence the synthesis technique selection.

### 2.1 Top-down method

The top-down process incorporates arc discharge, laser ablation, and acidic oxidation to break down the larger carbon resources into smaller components. This method generally employs carbon structures having sp^2^ hybridization that don’t have effective energy gaps or band gaps as precursor materials. The top-down method is very beneficial and effective for microsystem industries, but there are certain restrictions regarding the uniform size and shape distributions of CQDs, impurity, and costs (Table 1).

**TABLE 1:** Top-down method of carbon quantum dots synthesis.

Synthetic pathway	Precursors	Utilization	Ref	Advantages	Disadvantages
Laser ablation	Graphite powder and cement	Bioimaging	Sun et al. (2006)	(a) Scalable	(a) Expensive
	Nano-carbon materials	Biology and imaging	Li et al. (2011)	(b) easy and convenient synthesis process	(b) a large amount of carbon material is needed
	Carbon cloth	Cell bio-imaging	Cui et al. (2020)	(c) Ability to develop a range of nanostructures	(c) multi-step process
	Carbon microparticles	Cell labelling and visualization	Doñate-Buendía et al. (2020)		(d) harsh reaction conditions
	*Platanus* biomass	Engineering and biomedical imaging	Ren et al. (2019b)		(e) low control over size
					(f) low QY (quantum yield)
Arc discharge	Graphite electrodes	Optical activity	Biazar et al. (2018)	(a) Most obtainable method	(a) Required purification
	Graphite	energy technologies	Dey et al. (2014)		(b) Low QY (quantum yield)
	Carbon byproducts	Optoelectronics	Su et al. (2014)		(c) Harsh reaction conditions
Ultrasonication	Food waste	Optoelectronics, biomedical imaging, and plant seed germination and growth	Park et al. (2014)	(a) simple to use	(a) Low QY (quantum yield), (b) difficult to dope
	Cigarette ash	Cell imaging and cytotoxicity evaluation	Huang et al. (2018)	(b) Mild condition required for the experiment	
	Coke	Applied in light emitting devices	Feng and Zhang (2019)	(c) Cost- effective	
Electrochemical Method	Sodium citrate and urea	Selective sensing for mercury ion	Hou et al. (2015)	(a) no surface passivation is required	(a) allow only a few small precursors
	Amino acids	Cell imaging, fibre staining and specific sensitive detection towards ferric ion	Niu et al. (2017a)	(b) less expensive	(b) tedious purifying process
	Low-molecular-weight alcohols (ethanol)	Cell imaging	Deng et al. (2014)	(c) easy purification method	(c) Complex procedure
	Graphite rods	Applied in catalysis, bioscience and energy technology	Li et al. (2010)	(d) sustainable and environmentally friendly process	(d) Time-consuming process
					(e) tough to determine the CQDs concentration
Chemical oxidation	Activated carbon	Detecting metronidazole drugs using a chemiluminescence technique involving flow injection	Yan et al. (2014)	(a) Higher yield	(a) Poor quantum yield
	Coal	Applied in various optical devices and materials	Li et al. (2015)	(b) Highly pure	(b) Pollution of the environment
	Activated carbon	ECL (electrochemiluminescent) biosensing and bioimaging	Dong et al. (2010)		

#### 2.1.1 Laser ablation

This approach uses a high-energy laser pulse to illuminate the surface of the starting material. Consequently, it reaches a thermodynamic state characterized by incredibly high temperatures (>900°C) and pressures (75 KPa), leading to heating up quickly and condensing into a plasma state in which CQDs are formed by vapour crystallization. The first report of a laser ablation approach was made in 2006 by Sun and colleagues (Sun et al., 2006). The pulsed laser ablation method described by Ren X. et al., 2019 was utilized to produce N-doped micro-pore CQDs (NM-CQDs). NM-CQD was used for cellular staining and imaging and showed good internalization in various cells. Dual-beam laser ablation was described by Cui et al., 2020 as a means of producing carbon quantum dots (CQDs) from inexpensive carbon fabrics for utilization in bioimaging. The laser ablation method yields more PL emissions.

#### 2.1.2 Arc discharge

This process utilizes gas plasma generated in a sealed reactor inside an anodic electrode to reorganize carbon molecules as they break into smaller segments from bulk carbon sources. When the electric current is present, the reactor’s temperature can reach −3,700°C, resulting in massively energetic plasma. Carbon vapour accumulates in the cathode to produce CQDs (Su et al., 2014). When Xu and colleagues used the arch discharge approach first to isolate and purify a single-wall carbon nanotube, they unintentionally discovered fluorescent carbon quantum dots (Xu et al., 2004). The CQDs can exhibit blue-green, orange, or yellow fluorescence at a wavelength of 365 nm. Using HNO3, the carboxyl moiety (hydrophilic) was added to the CQD surface.

#### 2.1.3 Acidic oxidation

It was reported in 2014 that hetero-atom-doped CQDs may be synthesized on a wide scale via hydrothermal reduction and acidic oxidation. To begin, carbon nanoparticles were oxidized using a mixture of H_2_SO_4_, HNO_3_, and NaClO_3_. (Dong et al., 2010). Subsequently, the oxidized CQDs underwent hydrothermal reactions with sources of selenium, sulfur, and nitrogen. Precursors such as sodium hydrosulfide (NaHS), sodium selenide (NaHSe), and dimethyl formamide (DMF) have each been employed. The generated S-CQDs, Se-CQDs, and N-CQDs, have demonstrated tunable extended fluorescence life-span, improved good quantum yield (QY), and PL activity in contrast to pure CQDs (Yan et al., 2014). CQDs are also used to prepare drug delivery applications and electrochemical sensors once they are generated using the acidic oxidation process.

#### 2.1.4 Electrochemical

The electrochemical method, primarily introduced by Zhou and coworkers in 2007, was used to fabricate blue luminescent CQDs deriving out of multiwall carbon nanotubes. This method involves larger carbon precursors shredding into smaller parts through electrochemical oxidation, with a reference electrode. Zhang et al. have developed water-soluble CQDs exhibiting tunable luminescence (Hou et al., 2015). Deng et al., 2014 utilized this method to synthesize CQDs utilising low-molecular-weight alcohol. However, this technique needs surface passivation, and has a tedious purification process, making it the least frequently used technique.

#### 2.1.5 Ultrasonication

The CQDs preparation process is easy and economical with the use of ultrasonic technology. It creates waves of alternating high- and low-pressure causing tiny bubbles in liquid to evolve and burst. To achieve varied properties, researchers can alter the reaction time, ultrasonic power, and ratio of carbon source to solvent. CQDs have been produced using this technique from various carbon materials, such as graphite, MWCNTs, and carbon fibre. CQDs exhibit blue luminous emission and range in diameter from 1 to 5 nm (Park et al., 2014). The ultrasonic method can also be used to create heteroatom-doped CQDs (Huang et al., 2018). CQDs can also be made using other waste materials that contain carbon (Feng and Zhang, 2019). Food waste and ethanol can be combined to create water-soluble CQDs, which have several benefits including low cytotoxicity, strong photoluminescence, and high Photostability for *in vitro* bioimaging.

### 2.2 Bottom-up method

The top-down approach for CQDs synthesis utilizes techniques like combustion, hydrothermal/solvothermal, microwave irradiation, template, and pyrolysis (Table 2). The shape and size of CQDs depend on factors like precursor molecular structures, solvent, and reaction conditions. The reaction condition is important as it affects the reactants as well as the incredibly random nucleation and escalation process of the CQDs. This approach has great advantages in material chemistry, is easy to operate, has lower costs, and is easier for large-scale production. Precursors for CQD synthesis can be chemical or biological, with chemical precursors including glucose, citric acid, sucrose, and natural sources like Azadirachta indica leaves, rice husks, and aloe vera.

**TABLE 2:** Bottom-up method of carbon quantum dots synthesis.

Synthetic pathway	Precursors	Utilization	Reference	Advantages	Disadvantages
Hydrothermal	L-Ascorbic acid	Biological labelling, bioimaging, and diagnosis of disease	Zhang et al. (2010)	(a) Cheap	(a) Low yield
	Glucosamine hydrochloride	Biomedical, catalysis and chromatography	Yang et al. (2011)	(b) eco-friendly	(b) low purity
	Citric-acid, and ethylenediamine	Printing inks, biosensors for detecting Fe^3+^, and Bioimaging	Zhu et al. (2013)	(c) controllable	(c) Poor size control
	Phosphoric acid and folic acid	Biosensor for Pt^4+^ detection	Campos et al. (2016)	(d) non-toxic	
	Glycerol and (3-aminopropyl) triethoxysilane (APTS)	intracellular Cu^2+^ imaging in cells	Zou et al. (2016)		
	Citric acid and poly (ethylenimine)	Clinical and biochemical assays; morin detection in urine samples	Li et al. (2017a)		
	Tetraphenyl porphyrin and 1,2-ethanediamine (EDA)	Multicolour bioimaging and biosensors for Fe^3+^ ions detection	Wu et al. (2017)		
	Ethylene glycol, Folic acid	Biosensor for Hg^2+^ detection	Zhang and Chen (2014)		
Microwave	Diethylene glycol	antibacterial activity	Verma et al. (2020)	(a) Rapid, convenient and scalable	Poor size control
	PEG-200 and saccharide	Biological labelling and biosensors	Zhu et al. (2009)	(b) Inexpensive	
	Citric acid, branched polyethyleneimine	*In vivo* gene delivery	Pierrat et al. (2015)	(c) Environmentally friendly	
	Citric acid, urea	Screening of oxygen-states in CQDs	Dong et al. (2017)		
	Raw cashew gum	*In vivo* imaging	Pires et al. (2015)		
	Crab shell	Drug Delivery and Bioimaging	Yao et al. (2017)		
	Eggshell membrane	Sample detection and biotechnology	Wang et al. (2012)		
Combustion	Candle soot	Multicolor imaging	Liu et al. (2007)	(a) Large-scale synthesis	low quantum yield
				(b) Exhibited good PL intensity	
				(c) Simple, inexpensive, and eco-friendly method	
				(d) displayed good fluorescence without doping	
Template	F127	Bioimaging agents	Liu et al. (2009)	Simple to manipulate CQD’s size	(a) Time taking method
	Pluronic P123 and OMS (ordered mesoporous silica) SBA-15	Bioimaging	Yang et al. (2013)		(b) Cost -effective method
					(c) Limited quantum yield
					(d) Difficult steps
Pyrolysis	Durian peel waste	Supercapacitor	Praneerad et al. (2019)	(a) Rapid process	Broad size distribution
	Peanut shells	Multicolor cell imaging	Xue et al. (2016)	(b) Repeatable, practicable, and simple	
	Watermelon peel	Optical imaging probes and cell imaging	Zhou et al. (2012)		

#### 2.2.1 Combustion

The combustion method gained popularity for its ease of scaling up, accurate precursor molecule design, affordability, and environmentally friendly features to enhance the bottom-up methods for producing CQDs. The method of synthesizing CQDs using combustion was initially published by Liu et al., 2007. This process uses oxidative acid treatments to adjust the fluorescence characteristics, improve water solubility, and aggregate tiny carbon resources into CQDs. Liu and Coworkers clarified that the process of partially burning a candle with aluminium foil and refluxing it in a solution of nitric acid produced candle ashes, followed by dissoluting the candle ashes in a neutral medium, centrifuging the mixture, and using a dialysis technique to obtain Pure CQDs. Researchers have developed combustion methods for producing fluorescent CQDs, such as combusting citric acid, followed by surface functionalization with carboxyl groups through acetic acid moiety conjugation at elevated temperatures (Yang et al., 2023). The combustion approach synthesizes CQDs with poor QY but good fluorescence without doping.

#### 2.2.2 Hydrothermal

The hydrothermal process includes pouring the precursor materials into an aqueous media, then adding the mixture to a stainless-steel autoclave lined with Teflon and heating it to high pressure for many hours. Zhang et al., 2010 initially introduced the use of the hydrothermal method to prepare 2 nm-diameter fluorescent CQDs by employing L-ascorbic acid as a carbon precursor. The hydrothermal process, which has attracted major interest recently owing to its cost-effectiveness and environmental friendliness, produces CQDs from tiny molecules (amino acids, saccharides, sucrose, proteins, glucose, polymers, polyols, discarded peels or juice, etc.) in a single, environment friendly, and cost-effective way. Carbon quantum dots (CQDs) were made sustainably from biowaste obtained from banana peels incorporating a simple hydrothermal process by Lee et al. (Atchudan et al., 2020). When exposed to UV light (365 nm), these CQDs generate a strong blue fluorescence with a 20% quantum yield (QY). NS-CQDs (QY 53.19%) were also produced utilizing this process from L-lysine and thiourea to detect the concentration of picric acid in water. A solvothermal approach is also involved in synthesizing CQDs in which alcohol, ammonia, and other organic and inorganic solvents are employed in place of water (Zuo et al., 2015).

#### 2.2.3 Microwave

CQDs can be produced efficiently and economically via microwave synthesis, which employs electromagnetic radiation with a broad wavelength that ranges from 1 mm to 1 m (Ahmad et al., 2018). This radiation can provide sufficient energy to disrupt the chemical bonds of the reaction’s precursor components. In comparison to other techniques, this process is easy, straightforward, quick, and ecologically friendly, resulting in higher quantum yield for the CQDs formation. Zhu et al., 2009 and co-workers developed fluorescent CQDs having electrochemiluminescence abilities for the very first time by heating a clear aqueous solution of saccharides and PEG 200 in a 500 W microwave oven for 2–10 min utilizing this simple and affordable method.

#### 2.2.4 Pyrolysis

The pyrolysis technique is a thermal decomposition of a precursor, typically over 430°C, to create nanoscale colloidal particles. It offers practicality, repeatability, simplicity, and high quantum yield (QY). In the presence of a strong acid concentration and an alkali as a catalyst, the carbon precursor cleaves into colloidal nanoparticles. However, separating small precursors from raw materials is challenging. In 2009, Liu et al., 2009 developed a method for preparing CQDs using resol (source of Carbon) with surfactant-modified silica spheres. The CQDs were stable in a broad pH range and showed blue fluorescence. Xue et al., 2016 synthesized N-doped carbon quantum dots (CQDs) from peanut shell waste using an economical carbonization method, which shows excitation-dependent fluorescence emission. Praneerad et al., 2019 produced fluorescent CQDs from durian peel biomass waste, which was used for constructing a composite electrode with higher specific capacitance than a Carbon electrode.

#### 2.2.5 Template

Another commonly used approach for producing nano-sized CQDs is the template-assisted technique which provides higher quantum yields and even size distribution. In this method, CQDs are prepared via two steps. The first step involved calcination, which produced CQDs in the suitable silicon sphere or mesoporous template and the second step involved etching, which removed the supporting components. As a beneficial methodology for CQDs, this procedure is simple, accessible, inhibit particle agglomeration, appropriate for surface passivation, and regulates CQD size (Liu et al., 2009). Mesoporous silica spheres were used as nanoreactors by Zhong and colleagues in 2011 to generate carbon dots with exceptional luminescence characteristics. Templates can be made of hard elements like silica, metal-organic framework, layered host matrices, and zeolite, or soft elements like surfactants. Yang et al., 2013 created a novel soft-hard template technique for photoluminescent CQDs synthesized from organic molecules. The soft template in this report is Pluronic P123, while the hard template is OMS (ordered mesoporous silica) SBA-15. The drawback of the template method is that certain templates are challenging to extract from the CQDs, and the fluorescence activity of the CQDs could be impacted when the templates are removed by heating or acid-base etching. Besides all these techniques discussed here, CQDs can also be synthesized from porous organic polymer following a suitable method for various applications (Pan et al., 2019; Das et al., 2022a).

## 3 Characteristics of CQDs

Depending on whether sp^2^ carbon is present, the core-shell architectures of carbon quantum dots (CQDs) can have either an amorphous or graphitic crystalline structure. These tiny (2–3 nm) cores vary in size based on the synthesis method, precursors, and additional factors (Pathak et al., 2023; Yadav et al., 2023). Precursors, methods, and other factors are used to classify cores. The core structure of CQDs is ascertained using instrumental methods such as Nuclear magnetic resonance (NMR), Raman spectroscopy, X-ray diffraction (XRD), TEM, and SEM. X-ray photoelectron spectroscopy (XPS), elemental analysis (EA), and Fourier transform infrared (FT-IR) are further methods. NMR can also be employed to understand the interactions and relaxation studies of CQDs with various proteins (Jaipuria et al., 2012; 2022; Mondal et al., 2013; Dubey et al., 2016; Werbeck et al., 2020). Nitrogen sorption analysis is used to determine the surface area of carbon nanoparticles (Verma et al., 2020). Using zeta potential, the existence of functional groups on the outer layer is verified. The PL characteristics of CQD fluorescence emission arising out of the conjugated p domain are determined by the quantum-confinement effect from p-conjugated electrons within the sp^2^ atomic structure (Zhu et al., 2015). Fluorescence emission is caused by surface imperfections such as sp^2^ and sp^3^ hybridized carbon, and the size of CQDs influences the characteristics of PL (Luo et al., 2014; Zuo et al., 2015). Fluorescence emission and peak placement in CQDs can be impacted by surface imperfections and irregularities. CQDs can be created using the entire spectrum of sunlight (Ahmad et al., 2018). Moreover, photoexcited electron transfer is aided by the CQDs-based composites’ capacity to harvest light, which raises photocatalytic efficiency.

Carbon quantum dots, in contrast to semiconductor QDs, can exist in an amorphous form because of their predominant sp^2^ and sp^3^ molecular orbitals (Wang et al., 2014). Carbon QDs are often modified by surface passivation and functionalization with a particular organic ligand. As a result, several organic functional groups, including carboxylic, carbonyl, hydroxyl, and hydrocarbon, are capped on the surface (Cui et al., 2021). The carbon core is covalently linked to every member of this functional group. This effect makes CQD more soluble in water and makes preparing it for use in a variety of applications easier.

## 4 Properties of CQDs

CQDs are characterized by a “core-shell” nanostructure that is made up of surface functional groups and a nanoscale carbon core (Pourmadadi et al., 2023). Their various structural configurations and quantum confinement effect also affect their catalytic, optical, and electronic behaviours. These properties can further be modified utilizing surface passivation or doping the CQD (Yang et al., 2023). The bandgap and electronic structure are modified by doping and the presence of heteroatoms.

CQDs prepared out of various precursors, exhibit varying absorption spectra in different solvents (Wang et al., 2014). They have similar UV-visible absorption, with absorption peaks in the UV region ranging from 260 nm to 320 nm. The absorption peak in the 280–350 nm range is attributed to electronic transitions from C–O or C=O bonds to π* orbitals. The absorption peak in the 350–600 nm range is attributed to surface chemical moieties. Some CQDs display long-wavelength absorption ranging from 600 to 800 nm, originating out of the aromatic ring-containing structures (Cui et al., 2021). Surface modification or passivation can influence absorption properties. Carbon quantum dots (CQDs) can have their optical characteristics modified by surface passivation, functional groups, and heteroatom doping or co-doping (Yadav et al., 2023). The CQDs are shielded from impurity adherence and extra stability by the protective layer that surface passivation creates on their surface. When surface-passivating chemicals are added to CQDs, they exhibit increased quantum yields and fluorescence, making them highly optically active. Longer wavelength absorption can also be enhanced by surface passivation. Covalent bonds between functionalizing agents and CQDs can produce materials with exceptional photo reversibility, low toxicity, high stability, and strong biocompatibility (Gao et al., 2018). By modifying the π-π* energy level, the dopant modifies the bandgap, electrical structure, and optical characteristics of CQDs. Various processes (as mentioned in Section 2) can be utilized to create such heteroatom-doped CQDs, which have better photocatalytic activity, increased electron transfer, and higher QYs.

**FIGURE 2:**
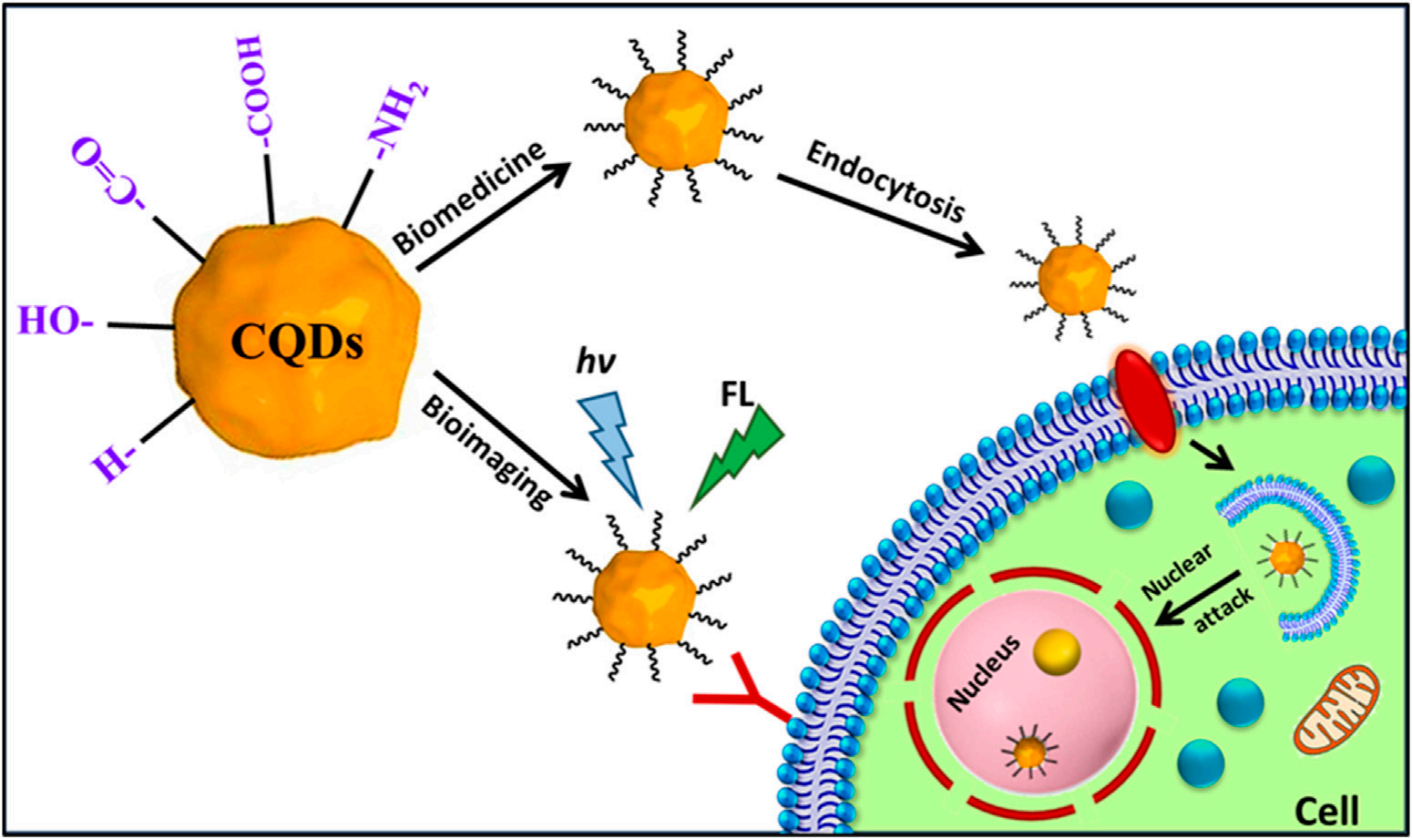
Schematic of the mechanism of actions of fluorescent carbon quantum dots inside the cells in bioimaging and biomedicine.

**FIGURE 3:**
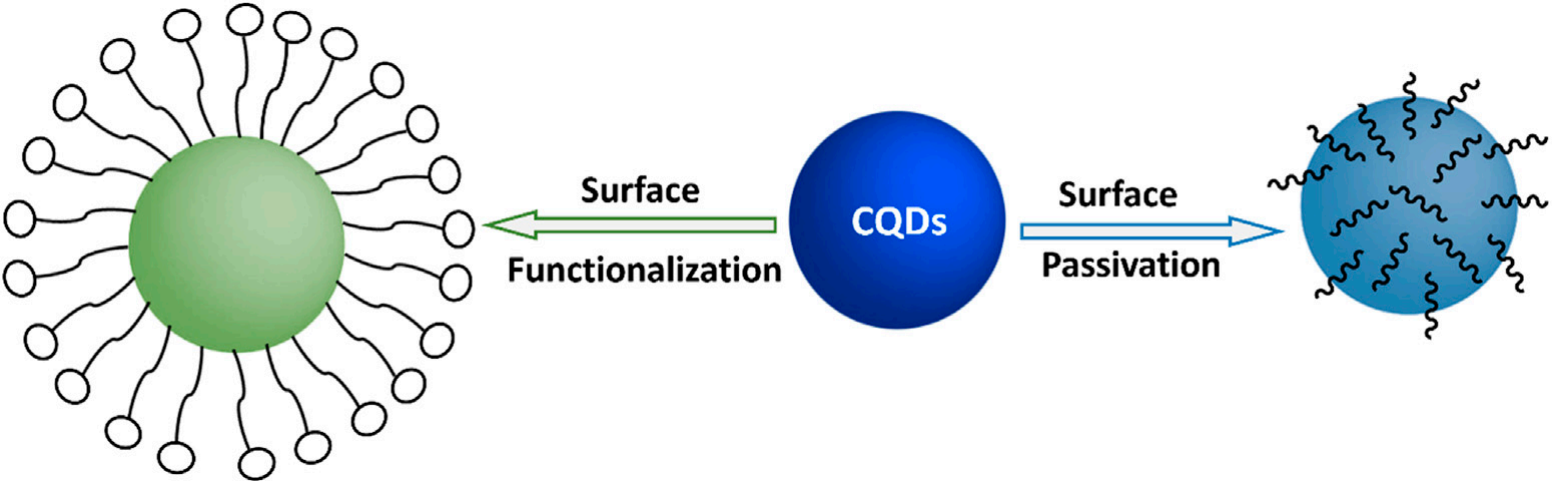
Schematic for surface functionalization and surface passivation on CQDs.

**FIGURE 4:**
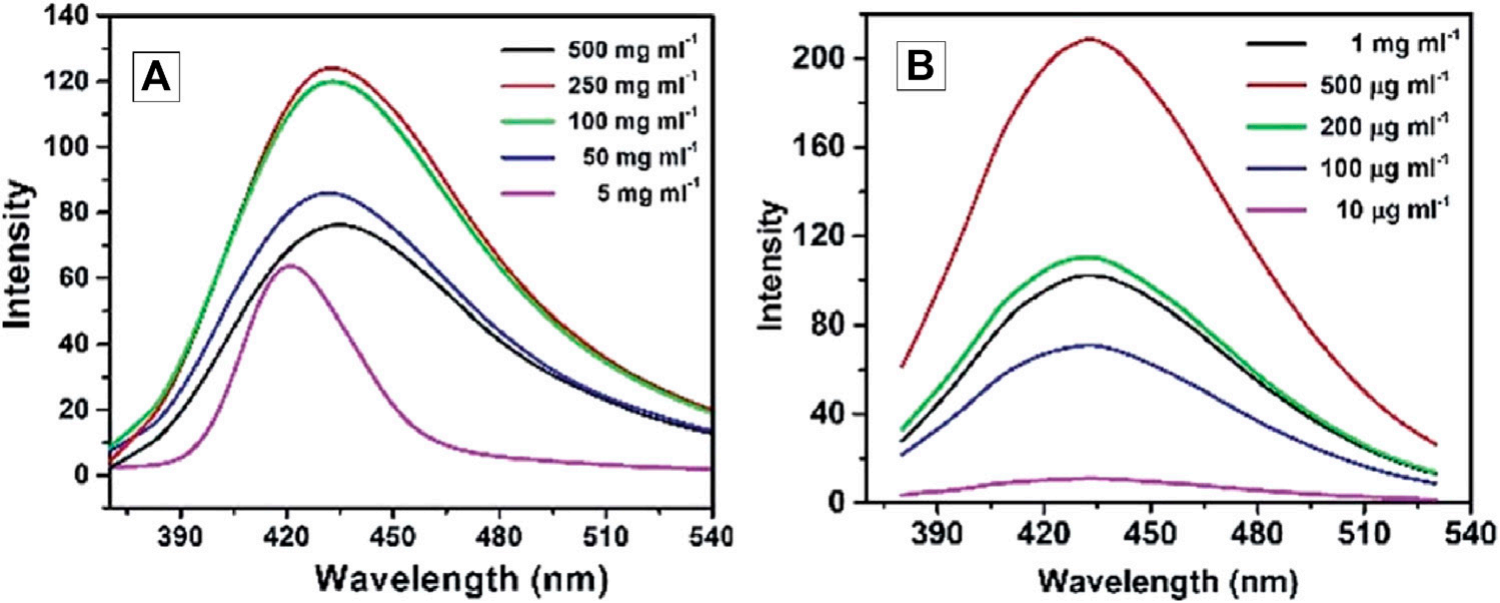
Fluorescence emission spectra (with excitation at 346 nm) of hydrophilic CQD **(A)** and hydrophobic CQD **(B)** prepared from sucrose and Octadecyl-amine/octadecene respectively. Reprinted (adapted) with permission from Mondal *et al.* (Mondal et al., 2015) Copyright (2015) Royal Society of Chemistry.

Fluorescent CQDs have great applications in sensing, bioimaging and biomedicines (Figure 2). There are various mechanisms to understand the fluorescence in CQDs (Yang et al., 2023). Fluorescence phenomena in CQDs are primarily produced by two mechanisms: (a) surface defects, surface passivation/functionalization (Figure 3), carbon core state, quantum size effect, and (b) band gaps of π-conjugated domains (sp^2^ hybridized) which resemble aromatic molecules incorporating certain energy band gaps regarding absorption and emission (Song et al., 2014). On the other hand, surface defects arise in CQD owing to the unsymmetrical arrangement of sp^2^ and sp^3^ carbon atoms, and the presence of hetero atoms like N, B, P, or S. Due to the surface defect, the solid host creates an environment like aromatic molecules resulting in absorbing UV light to exhibit various colour emissions. The two emission forms are seen in CQDs: excitation-independent emission because of the highly ordered graphitic structure, and excitation-dependent emission (tunable emission) because of different emission sites and particle size distribution (Figure 4). In contrast to conventional organic dyes, CQDs have high photostability and steady fluorescence.

**FIGURE 5:**
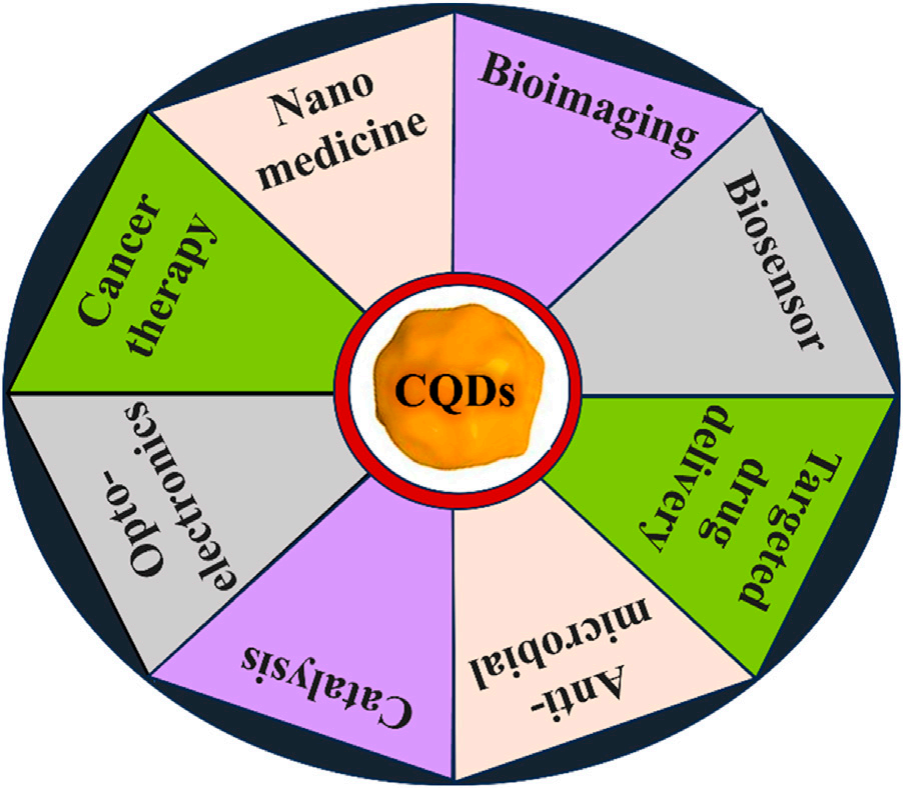
Various applications of CQDs. We discussed recent advancements in bioimaging and biomedical applications using CQDs in our review.

Recent studies have explored the photoluminescence (PL) emission in carbon quantum dots (CQDs), which has gained interest in photocatalysis with other fields (Yang et al., 2023). PL emission wavelengths are larger than the excitation wavelength and can be attributed to band-gap transitions incorporated in conjugated π-domains or deficiencies in graphene structures. Research has shown that excitation-dependent emission of fluorescent CQDs with surface modifications and one-step thermal treatment of 4-amino antipyrine emit excitation-dependent PL emission in 525–660 nm. The investigation looks at the PL emission of CQD, with a particular emphasis on size modification. CQDs were created by an electrochemical method assisted by alkali, and their diameters ranged from 1.2 to 3.8 nm (Hasanzadeh et al., 2021). The PL characteristics fluctuated with particle size, as revealed by optical views, suggesting a highly reliant HOMO-LUMO gap (Zhu et al., 2015). Particles with sizes smaller than 3 nm are more likely to emit visible spectrum light because the band gap narrows with increasing particle size. PL emission from CQD produced from alkyl-gallates is independent of size. In this review, we will discuss the recent advancements in bioimaging and biomedical applications employing all these features and various modifications of CQDs (Figure 5).

## 5 CQDs in bioimaging

Bioimaging enables real-time, non-invasive imaging of biological phenomena. It is a technique of utmost importance in healthcare units for the diagnosis of human health diseases. The intrinsic fluorescence property of CQDs, great stability, and great advantage of modification with various functional groups offer a suitable candidate for bioimaging (Bhunia et al., 2013; Luo et al., 2014; Liu et al., 2020b; Su et al., 2020). In addition, multi-wave-length emissions, excellent photostability, resistance to photobleaching, and quick and easy methods of preparation, establish CQDs to be the fluorescent probes of the next-generation for *in vitro* and *in vivo* imaging (Wegner and Hildebrandt, 2015; Wang et al., 2022; Gedda et al., 2023). Other conventional quantum dots like metal quantum dots and organic dyes are toxic, mostly prohibiting their uses in bioimaging (Su et al., 2020; Wang et al., 2022). Three-dimensional visualization of the biological subcellular compartments as well as tissues and organisms can be obtained with various biocompatible carbon quantum dots (Table 3).

**TABLE 3:** Bioimaging applications of various carbon quantum dots.

Type of CQDs	Precursors	Synthesis method	Ref
CQDs	Wheat straw and bamboo	Hydrothermal	Huang et al. (2019)
CQDs	Graphite powder, Cement, Poly-(propionyl ethylenimine-*co*-ethylenimine)	Microwave-assisted pyrolysis	Cao et al. (2007)
Pure red emissive CQDs	Urea and citric acid	Solvothermal	Zhang et al. (2022)
N, S, P-CQDs	Thiamine pyrophosphate (ThPP)	Hydrothermal	Nasrin et al. (2020)
CQDs	Citric acid and para-phenylenediamine	Hydrothermal	Huo et al. (2022)
N-CQDs	Tetraphenyl porphyrin	Hydrothermal	Wu et al. (2017)
CQDs	Aconitic acid	Hydrothermal	Qian et al. (2018)
CQD	Maltose and NaOH	Microwave-assisted	Shereema et al. (2015)
		Method	
L-CQDs	Citric acid and ethanediamine	Hydrothermal	Xue et al. (2019)
CQDs	Walnut shells	Carbonization	Cheng et al. (2017)
N, Cl- CQDs	Urea and choline chloride-glycerine deep eutectic solvent	Microwave-assisted method	Wang et al. (2018)
CQDs	*Eleocharis dulcis*	Hydrothermal	Bao et al. (2018a)
N, P-CQDs	H_3_PO_4_, Cyanobacteria and C_2_H_8_N_2_	Hydrothermal	Wang et al. (2021)
N-CQDs	Guanidinium chloride and citric acid	Pyrolysis method	Mingcong et al. (2017)
CQDs	Banana peel	Hydrothermal	Atchudan et al. (2020)
Si-CQDs	Hydroquinone	Solvothermal	Qian et al. (2014)
N, P-CQDs	Ganoderma lucidum	Hydrothermal	Tu et al. (2020)
CQDs	Cynodon dactylon	Microwave-assisted method	Gurung et al. (2023)

**FIGURE 6:**
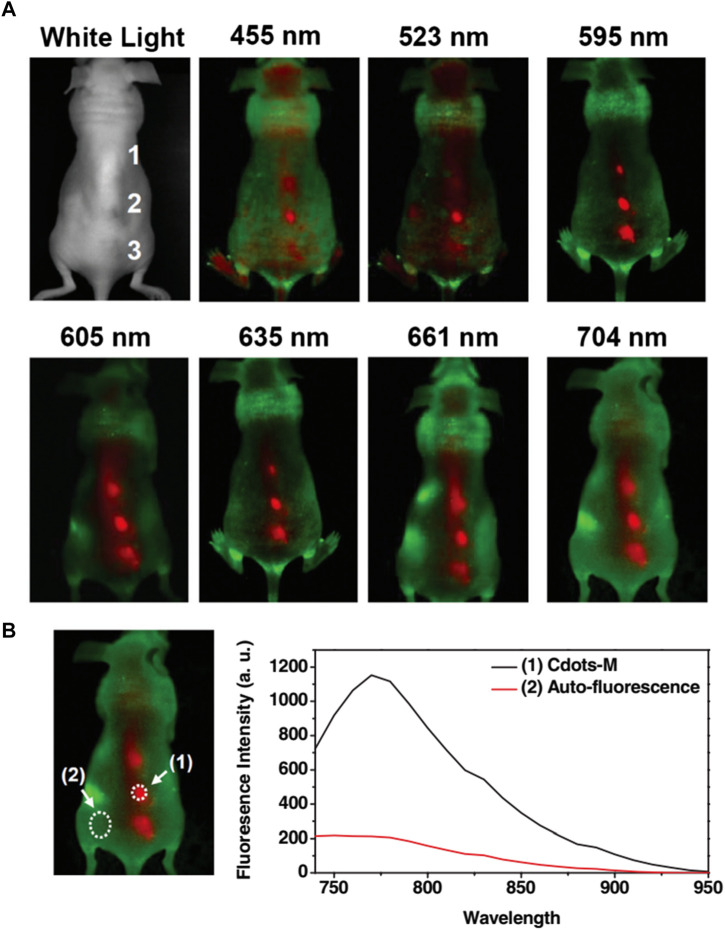
**(A)** Fluorescence imaging *in vivo*. *In vivo* fluorescence photos of a mouse administered with CQD. The pictures were captured at 455, 523, 595, 605, 635, 661, and 704 nm at different excitation wavelengths. Tissue autofluorescence and CQD fluorescent signals are shown in red and green, respectively. **(B)** The spectral picture obtained with NIR (704 nm) excitation has a signal-to-background separation. The background of tissue autofluorescence was clearly distinguished from the CQD fluorescence. High radioactivity of I^125^ was found in the urine and faeces of the mouse, indicating that both renal and faecal excretions may have contributed to the CQD’s clearance. Reprinted with permission from Tao et al. (2012) Reprinted (adapted) with permission from Liu et al*.* Copyright (2012) Wiley.

Biocompatible CQDs can readily penetrate various cells through endocytosis, a macropinocytosis-like cell-penetration mechanism based on the shape, surface functionalization, and type of the cells (Magesh et al., 2022; Wang et al., 2022). Bioimaging of such cellular compartments and organelles can lead to a broader understanding of early symptoms and progression of different diseases like Alzheimer’s, Parkinson’s, diabetes, cancers, and many others (Kabanov and Gendelman, 2007; Guerrero et al., 2021). CQD can be employed to understand the interactions with various proteins and track the changes through Bioimaging (Bhunia et al., 2013; Luo et al., 2013; Wang et al., 2022). The unique properties of CQDs, such as their outstanding photostability, low cytotoxicity, conspicuous biocompatibility, and multicolour emission profile, make them a prime option for fluorescence imaging (Luo et al., 2014). Up until recently, almost all CQDs have essentially labelled the cytoplasm and cell membrane without any special alterations. CQDs were used for intracellular imaging with HeLa cells, MCF-7 cells, Caco-2 cells, HepG2 cells, PC12 cells, lung cancer cells, and pancreas stem cells (Li et al., 2018; Xia et al., 2019; Shi et al., 2020; Tian et al., 2020; El-brolsy et al., 2022; Havrdová et al., 2022). Besides, researchers have systematically tracked the biodistribution of CQD in mice cells through *in vivo* imaging (Shi et al., 2020; Tian et al., 2020). Tao et al., 2012 performed *in vivo* fluorescence imaging investigations (Figure 6) on CQD-M derived from MWNTs. The researchers labelled CQD-M I^125^. When the radiolabeling stability was examined in mouse plasma, the amount of I^125^ detachment from CQDs was found to be satisfactory. The biodistribution and blood radioactivity levels were used to measure the pharmacokinetics of CQDs. According to the study, following intravenous injection, CQDs were mostly collected in the liver and spleen. Early on, there was a significant level of kidney uptake of CQDs, indicating that they might pass through the glomerulus and be eliminated by urine. This study indicates that both renal and faecal excretion could lead to the clearance of CQDs (Tao et al., 2012).

Using CQDs in polymer matrices (Carbonized Polymer Dot or CPD) is an additional new field of study with a broad range of possible applications (Feng et al., 2021). CQDs with polyethyleneimine (PEI) functionalization display tunable fluorescence with multiple wavelength emissions (Chen et al., 2019). The ternary nano-assembly of CD-PEI/Au-PEI/pDNA renders an effective transfecting agent as observed from a fluorescence microscope (Kim et al., 2013). CQD is functionalised with quaternary ammonium groups employed in L929 and NIH/3T3 cell lines of healthy mice and imaged with fluorescence microscopy (Havrdová et al., 2021). This study reveals the intranuclear uptake of the functionalised CQDs utilising their intrinsic fluorescence properties. Photoacoustic (PA) imaging and near-infrared fluorescence are displayed by large amino acid mimicking (LAAM) CQDs synthesised from 1,4,5,8-tetraminoanthraquinone and citric acid (Li et al., 2020). LAAM CQDs can selectively image tumours. CQDs prepared from o-phenylenediamine and terephthalic acid applied for near-infrared (NIR) bioimaging (Ding et al., 2020; Moniruzzaman et al., 2022; Marković et al., 2023). A donor-acceptor (D-π-A) structural approach was introduced for the synthesis of this CQD. CQD-PEG was developed by choosing polyethylene glycol as a passivating agent for the increment in functionality and photoluminescence (PL) properties of CQDs (Peng et al., 2020). CQD-PEG were exceptionally photoresponsive and photoluminescent after the surface modification. PEG passivated CQDs were initially used to stain Caco 2 cells for cellular bioimaging, rendering CQD a potential fluorescent label of the cells. Red emissive CQDs (absorption: 400–750 nm) were prepared using polythiophene phenyl propionic acid (Ge et al., 2015; Liu et al., 2020a; Ding et al., 2020). The red emissive CQDs exhibit a high photothermal conversion efficiency (η ∼ 38.5%) and a strong photoacoustic response. These special qualities allow the red emissive CQDs to be utilised as photoacoustic, multifunctional fluorescent, and biomedicines (discussed in the next section). Most CQDs are synthesized from graphite-based materials (GQD) that exhibit characteristics of graphene and can be utilized for bioimaging applications (Kortel et al., 2020). In the past, some techniques have been used to obtain mitochondrial imaging, such as labelling tumour cells with aptamer AS1411 and causing CQDs to collect at the mitochondrial and lysosomal sites. For imaging the nucleus, graphene-based CQD-PEI (Polyethyleneamine) was used (Tian et al., 2020). Following the addition of CQD/hydrogel loaded without and with 5-fluorouracil, bioimaging of A549 cells was carried out. CQDs were functionalized after being produced with citric acid using hydrothermal carbonization involving RGERPPR and maleimide-polyethylene glycol-amino succinimide succinate (Mal-PEG-NHS) (Gao et al., 2018). The resulting CQD was utilized for bioimaging. Gadolinium-encapsulated carbon dots can exhibit high T1 relaxivity (16.0 × 10^−3^ M^-1^ S^−1^, 7T) and intense fluorescence, enabling an imaging probe with intrinsically dual-mode (Jiao et al., 2022). The increased permeability and retention effect of these carbon dots helps them to accumulate readily in tumours and the unbound Gadolinium excretes from the host via the renal system.

**FIGURE 7:**
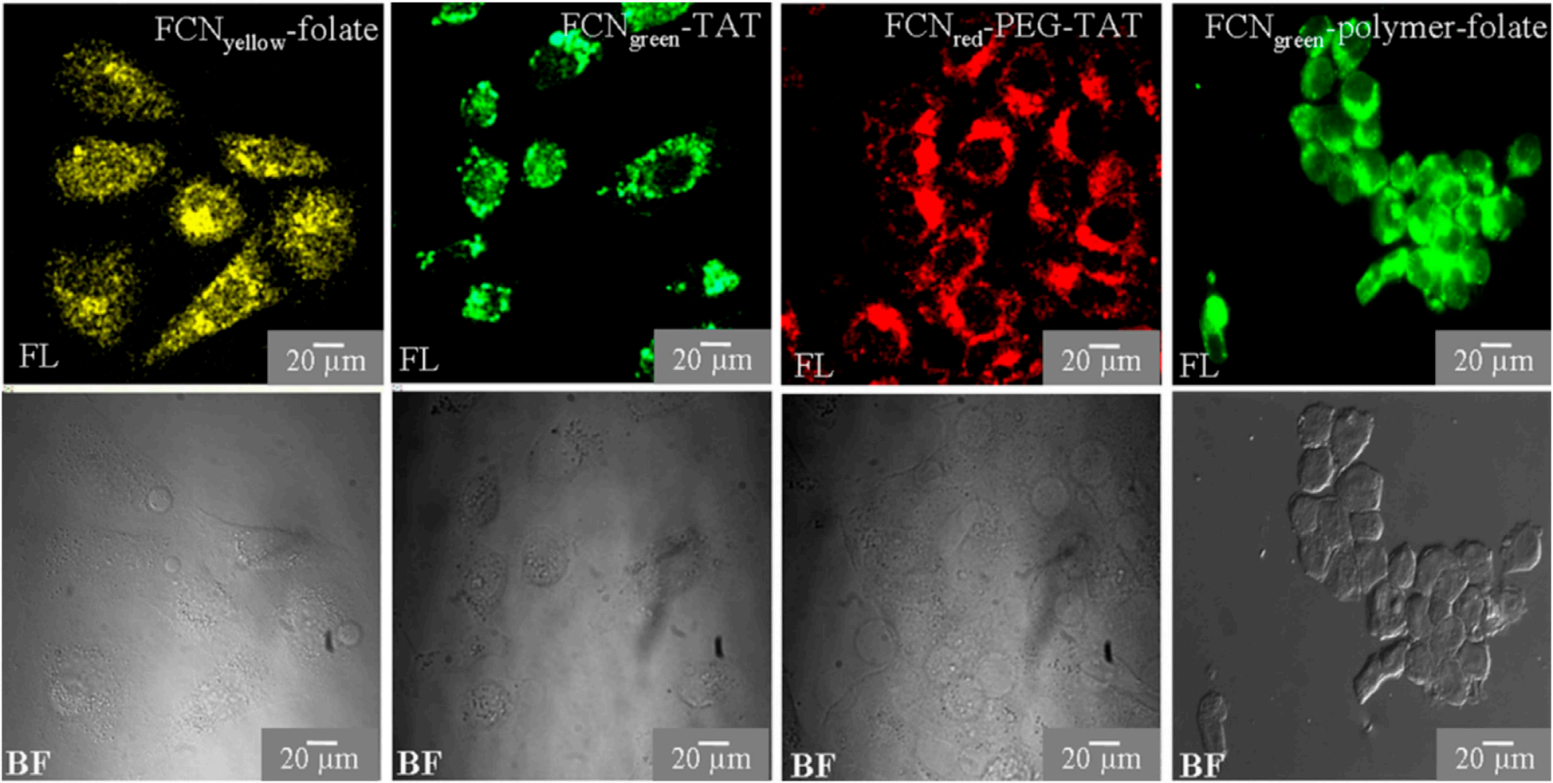
CQD as a fluorescent marker of cells. HeLa cells are cultured in CQDs for three to 6 hours, after which the labelled cells are observed under a fluorescent microscope. Using a confocal or Apotome microscope, cells are observed in bright field (BF) and fluorescence (FL) modes. Reprinted (adapted) with permission from Bhunia et al., 2013 Copyright (2013) Nature.

Hydrophilic and hydrophobic CQDs can be obtained with the preferred synthetic routes (Section 2) which is not common for other metal quantum dots for biological applications. Hydrophobic CQDs in parallel to hydrophilic CQDs offer a wide range of applications for the study of membrane proteins and other biological systems. The application of functionalized CQDs as fluorescent cell labels were studied by Bhunia et al., 2013 (Figure 7). They discovered that the CQDs may be imaged with TAT peptide- or folate-functionalization utilizing a standard microscope and can be labelled in one to 2 hours. Because of the low surface charge and tiny hydrodynamic diameter, CQDs have poor non-specific binding to cells which gets improved by this functionalization. The cellular and *in vivo* imaging applications of fluorescent CQDs are demonstrated by the fact that they are non-toxic at dosages higher than typical concentrations as concluded from MTT assays (Bhunia et al., 2013).

**FIGURE 8:**
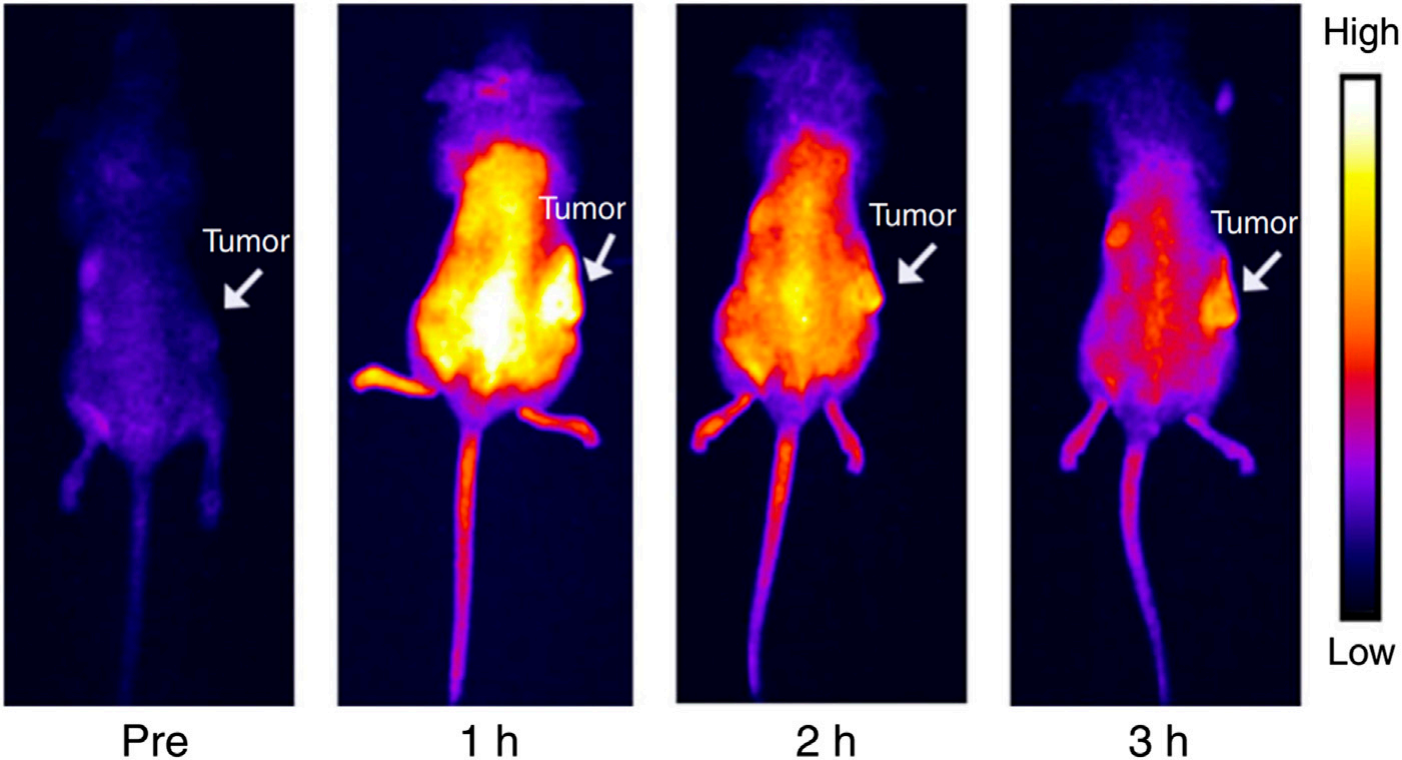
NIR emitting CQDs for *in vivo* imaging: Mouse bodies were imaged using NIR fluorescence at different time intervals following intravenous administration of CQDs (0.2 mL, 1000 μg mL^−1^). Reprinted (adapted) with permission from Bao X. et al., 2018 Copyright (2018) Nature.

Hetero-atom-doped CQDs offer a wide range of wavelengths with multicolour fluorescence properties, increasing the stability and perfect for real-time cellular bioimaging (Sekar et al., 2022; Khoshnood et al., 2023; Mousa et al., 2023). In addition, when CQDs are co-doped with two heteroatoms from Nitrogen, Boron, or Phosphorus, the resulting CQD becomes more biocompatible with a much higher quantum yield (Atchudan et al., 2020; Kalaiyarasan et al., 2020; Tummala et al., 2021; Sekar et al., 2022). When o-phenylenediamine was treated with boric acid, the hydrothermal reaction yielded N, B-CQDs which is yellow fluorescent (Wei et al., 2020). N, B-CQDs were incorporated with HeLa cells to obtain fluorescence bioimaging. N or B plays a significant role in electrical modification and a remodelled surface pattern in co-doping that advances intense radiation features in N and B-CQDs (Wei et al., 2020). Fluorescent N, P-CQD derived from hydrothermal synthesis of *Ganoderma lucidum* was utilised for *in vivo* imaging (Tu et al., 2020). Photoluminescent CQDs produced from alginate have enormous potential as bioimaging probes (Zhou et al., 2016). The functional effect of various element dopants on CQDs in generating multiwavelength emission is still under investigation. Bao et al. looked at the *in vivo* biodistribution of the CQDs utilizing NIR FL imaging of mice without and with tumours to determine if it would be feasible to use CQDs for tumour diagnosis and treatment (Bao X. et al., 2018). Following intravenous infusion of CQDs into mice with H22 tumours, each mouse’s entire body progressively displayed intense near-infrared fluorescence. The whole-body NIR fluorescence intensity had significantly dropped 3 hours after injection, and the tumour area’s NIR fluorescence signal stood out sharply from the surrounding tissues (Figure 8) (Bao X. et al., 2018). Graphene-based CQDs can also be utilized for red and NIR fluorescence bioimaging.

The need for efficient multimodal imaging probes is growing, combining various imaging modalities like magnetic resonance (MRI), photoacoustic (PA), and imaging using computed tomography (CT) (Prabhuswamimath, 2022; Rajasekar et al., 2022; Kanungo et al., 2023; Latha et al., 2023). PA imaging is a hybrid method of imaging that combines optical and ultrasonic modalities for deep tissue penetration and great spatial resolution (Rajasekar et al., 2022; Latha et al., 2023). CQDs exhibit substantial absorption coefficients in the red to near-infrared spectrum when used as PA contrast agents and can transform light into heat (Ge et al., 2015; Prabhuswamimath, 2022). Doping MRI/CT probes into CQDs allows for the formation of further multimodal CQDs as discussed in this section earlier. The spatial resolution and the limited penetration depth with multi-modal CQDs are still under investigation for application in PA, MRI, and CT imaging (Louie, 2010; Zheng et al., 2016; Morato et al., 2021). CQDs have a lot of potential applications in biological imaging as optical nanoprobes in the future (Wang et al., 2022). These findings suggested that cell imaging as an effective technique for *in vivo* imaging has a bright future. Subsequent research endeavours ought to concentrate on augmenting the QY of CQDs and generating structurally, compositionally, and geometrically well-defined CQDs. When CQD development is unrestricted by size, it can reach incredible ranges of applications.

## 6 CQDs in biomedicines

Nanotechnology is gaining attention in biomedical applications, particularly in nano-drug delivery systems and nano-cancer imaging (NCI) (Koutsogiannis et al., 2020). In the case of CQDs, the nano-drug delivery methods provide efficient delivery at a fixed rate and time, whilst the latter has the advantage of CQDs providing high sensitivity, improved contrast, and high precision (Azam et al., 2021; Cui et al., 2021; Zhu et al., 2022). The unique photophysical and physicochemical properties of CQDs along with their biocompatibility and facile synthetic route of preparation render CQDs an enticing nanomaterial candidate for biomedical application (Arshad et al., 2020; Azam et al., 2021; Adam et al., 2022). So CQDs have great applications in clinical research. The use of CQDs in drug delivery, gene therapy, and combatting the recent COVID-19 pandemic has been discussed below (Table 4).

**TABLE 4:** Biomedicinal applications of different carbon quantum dots.

Type of CQDs	Precursors	Synthesis method	Application	Ref
N-CQDs	Osmanthus leaves/Tea Leaves/Milk vetch	Hydrothermal	Antibacterial activity against *E. coli* and *S. aureus*	Ma et al. (2020)
CQD_Spds_	Spermidine powder	Pyrolysis	Bacterial keratitis treatment	Jian et al. (2017)
N, S-CQDs	Amino acids (Arginine, Lysine, Histidine, Cysteine, and Methionine)	microwave irradiation	Hemolysis and blood clotting tests	Sahiner et al. (2019)
Levofloxacin-CQDs	Levofloxacin hydrochloride	hydrothermal Hethod	Poor drug resistance and significant antibacterial activities	Wu et al. (2022)
Quaternized CQDs	Glucose and DDA (dimethyl diallyl ammonium chloride)	Carbonization	Wound treatment for multiple bacterial infections	Zhao et al. (2022)
N-CQDs	Diethylenetriamine and glucose	Hydrothermal	Antibacterial properties against *Staphylococcus*	Zhao et al. (2019)
CD-PEI-IBAm	Polyethylenimine (PEI) and glycerol and isobutyric amide	Pyrolysis	Biomedical treatment	Yin et al. (2013)
NHF-CQDs	*N*-hydroxy phthalimide	Pyrolysis	Treatment for metastatic breast cancer	Tiron et al. (2020)
Cur-CQDs	Curcumin	Pyrolysis	Significant antimicrobial efficacy against Enterovirus 71 (EV71) infection	Lin et al. (2019)
CQDs	κ-carrageenan and folic acid	Hydrothermal process	Nano-vehicle for cancer cell targeting, biomedical research, and anticancer drugs	Das et al. (2019)
QCQD	Diallyl dimethylammonium chloride and 2,3-epoxypropyltrimethylammonium chloride	Solvothermal	Therapy for pneumonia in mice infected with MRSA	Zhao et al. (2020)
CQDs	Gallic acid (GA), citric acid (CA), ethane diamine (EDA)	Microwave assisted method	Antitumor treatment	Lu et al. (2019b)
CQDs	Silk fibroin	Microwave assisted method	Biomedical	Ko et al. (2017)
CQD/Ag NPs	Cow milk	Hydrothermal	Antibacterial activity	Han et al. (2015)
CQDs	Diethylene glycol and amine	Microwave-assisted synthesis	Antibacterial activity	Verma et al. (2020)
CQDs	Sodium citrate dehydrate, Urea	Carbonization	Intracellular drug delivery	Sun et al. (2020)
CQDs	Ammonium citrate	Pyrolysis	Microbicide to treat MRSA infection	Li et al. (2016b)

### 6.1 Drug delivery

**FIGURE 9:**
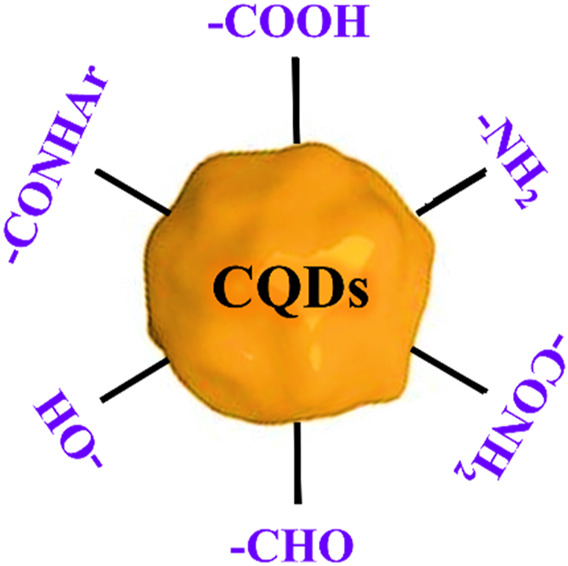
Schematic of different functional moieties on CQD which can be synthesized via any preferred methods as discussed in Section 2.

Like other nanomaterials, CQD-based drug delivery involves drugs onto CQDs through binding or adsorption, for site-specific delivery of the drugs with minimal side effects (Lim et al., 2015; Zhu et al., 2015; Guerrero et al., 2021). The bond between CQD and the drug gets cleaved in the CQD-drug complex in an acidic environment of the diseased site or other stimulating factors (Li et al., 2020). This allows the controlled release of the drug to specific sites. Free CQDs easily get excreted through the renal or hepatobiliary system afterwards (Tian et al., 2020). The controllable surface modification for varying functions, small size, low cost, biocompatibility, and almost no side effects make CQDs an easy choice for the drug target (Figure 9) (Zhang et al., 2018; Lu et al., 2019a). To date, several anticancer and antibacterial drugs have been delivered successfully utilising various modified CQDs (Verma et al., 2020; Mahani et al., 2021).

**FIGURE 10:**
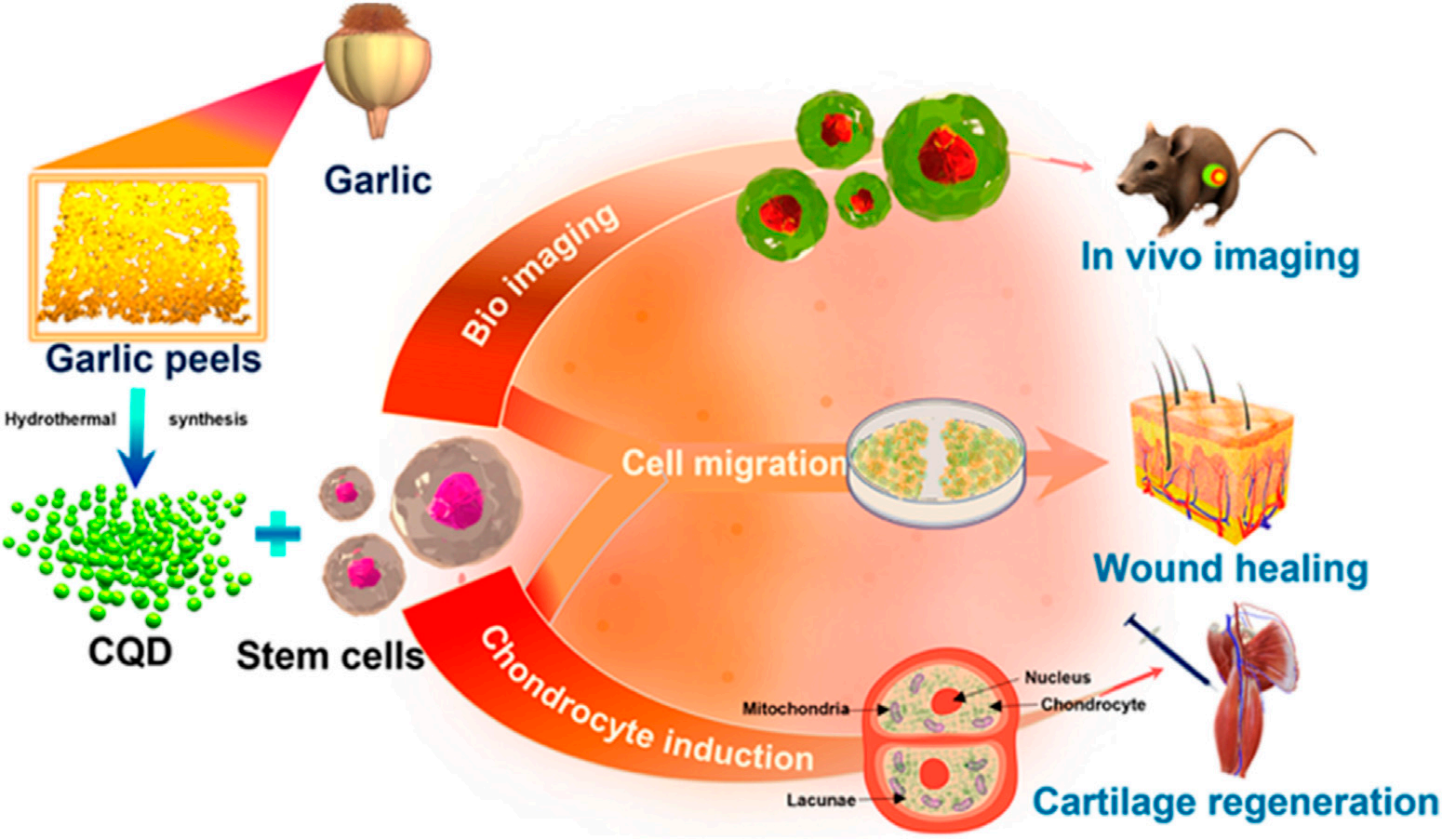
CQDs as a biogenic precursor for stem cell biology, demonstrating their ability to mesenchymal stem cell imaging without cytotoxicity and generate reactive oxygen species. Reprinted (adapted) with permission from Majood et al., 2023 (ACS Appl. Mater. Interfaces 2023, 15, 16, 19997–20011) Copyright (2023) ACS.

CQDs play a significant part in both the diagnosis and treatment of cancer-like diseases (Palashuddin Sk et al., 2015; Wang et al., 2015; Zhao and Zhu, 2016; Gao et al., 2017; Ghosh et al., 2019a). Doxorubicin is a common chemotherapy medication that has been licensed by the FDA (Li S. et al., 2016). Doxorubicin can be combined using enzymes associated with DNA from malignant cells to speed up DNA base pair intercalation in tumours and inhibit the growth of cancer cells, making it a typical first-line treatment for a variety of malignancies (Tacar et al., 2013). A combination of CQDs and doxorubicin, cisplatin, and docetaxel-like cancer drugs has been developed that exhibits considerable tumour targeting, improved anti-tumour effectiveness, and minimal side effects (Yang et al., 2016; Mahani et al., 2021; Mahani et al., 2021; Sawpari et al., 2023). Carbonized Polymer Dot or CPD has various medical applications (Zhu et al., 2015). CQD/polymer composites exhibit self-release patterns and have important biomedical utilisations in treating cancer (Zhao and Zhu, 2016; Adam et al., 2022; Soumya et al., 2023). They are used in drug delivery systems such as insulin-smart nanocarriers and chemotherapeutic medicines (Devi et al., 2019; Alaghmandfard et al., 2021). The N-doped carbon dots (FN-CQDs) with folic acid are endocytosed specifically (specific cellular absorption rate >93.40%) and remain in autophagic vacuoles in cancer cells for an extended period (Khoshnood et al., 2023). Released FN-CQDs have been proven to effectively kill tumour cells and to be effective against 26 different types of tumour cells by activating the extrinsic and intrinsic apoptotic signalling pathways. Selective tumour targeting of the human glioblastoma cell line (U87MG) was successful in utilizing surface charge modulation of CQD-Doxorubicin incorporating a coating of octylamine-modified polyacrylic acid (cRGD-PAA-OA) and cRGD (Gao et al., 2018). Zhou et. al. produced intensely red emissive CQDs with several coupled α-carboxyl and amino groups to improve drug delivery efficiency and tumour-specific imaging (Liu et al., 2020a; Ding et al., 2020). These CQDs could target tumours such as gliomas because of the large neutral amino acid transporter 1’s multivalent interaction (Liu et al., 2018). Consequently, the CQDs could likewise be used to treat brain tumours by fluorescence/PA imaging while combining with topotecan hydrochloride (Huang et al., 2013; Huang et al., 2013; Li S. et al., 2016; Deng et al., 2018; Li et al., 2020). The schematic representation of CQD-mediated drug delivery is shown in Figure 9 above. CQD on the Porous polycaprolactone (PCL) matrix strengthens the bioactivity for biomineralization and can be used for bone tissue engineering (Luo et al., 2014). Stem cell biology is a significant advancement in biomedicine, with researchers exploring the use of untapped stem cells. Mukherjee et al. propose a strategy using biogenic carbon quantum dots (CQDs) out of garlic peels as a biogenic precursor (Figure 10) (Majood et al., 2023). These CQDs can image mesenchymal stem cells without cytotoxicity and can form reactive oxygen species (ROS) to influence stem cell migration and chondrocyte differentiation without chondrogenic induction factors. The study suggests garlic peel-generated CQDs as a major advancement in stem cell biology.

**FIGURE 11:**
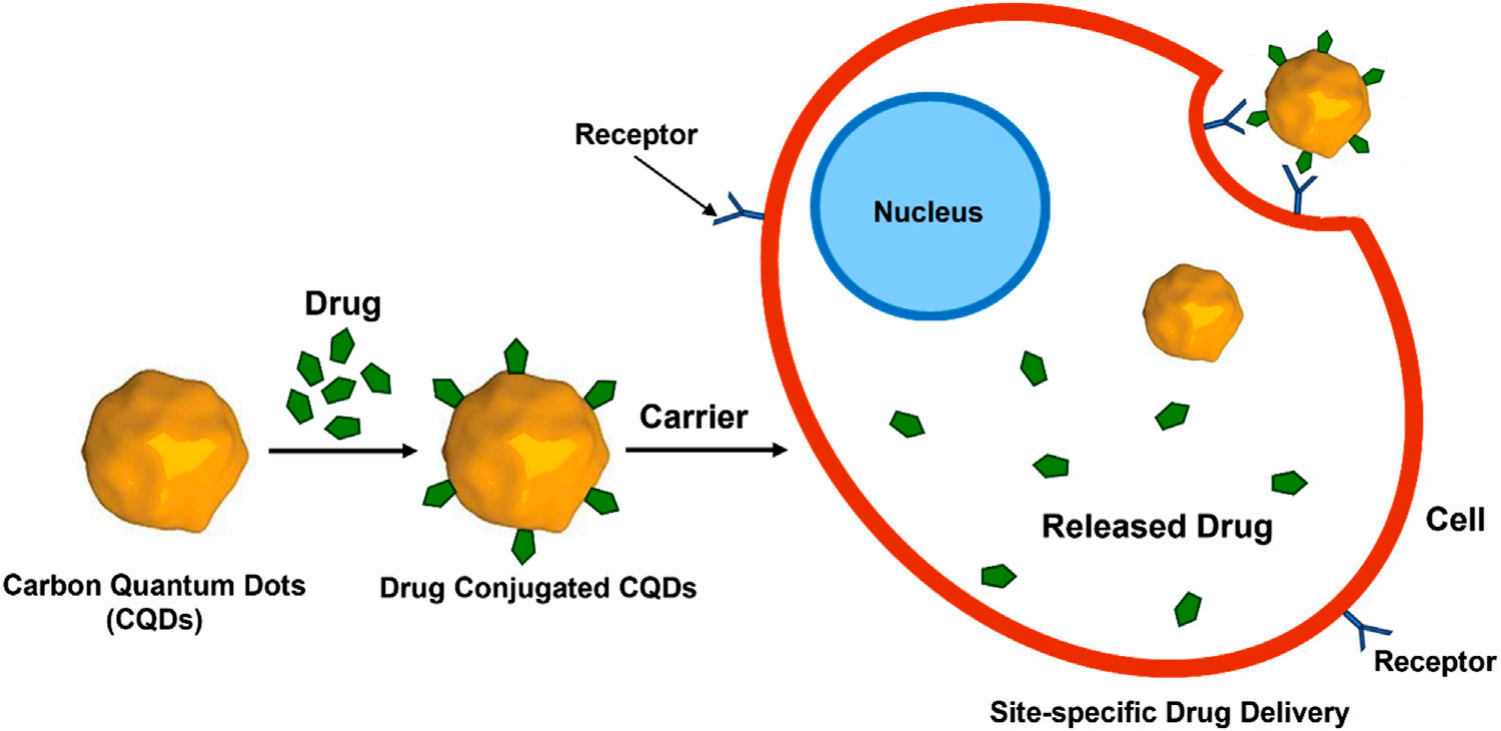
CQD-mediated drug delivery to the cells. Drug molecules are conjugated with CQDs followed by binding to the receptor on the cell surface and release of the drug molecules inside the cells.

PEGylation is a common surface functionalization approach used in the CQDs assembly; (CQDs-Pt (IV) @PEG-(PAH/DMMA)), a cisplatin-based platform that stimulates the electrostatic repulsion mechanism that releases positively charged CQDs-Pt (IV*)* (Feng et al., 2016)*. In-vivo* trials highlight the potential of CQDs for excellent tumour suppression efficacy and few side effects by emphasizing the efficiency of CQDs for transporting cisplatin-based drugs to the site-specific organs for maximal therapeutic effect. Cur-CPDs, CQDs prepared from curcumin, have anti-cancer, and antibacterial, anti-inflammatory, antioxidant properties (Lin et al., 2019). Insulin-smart nanocarriers, a self-release pattern developed by adding insulin to CQD/polymer hydrogels that contain phenyl-boronic acid are widely used in biomedical applications (Adam et al., 2022). An agarose-poly (vinyl alcohol) copolymer and CQDs as a cross-linker were used to generate a pH-responsive hydrogel nanocomposite, and this biodegradable nanocomposite released the antibacterial medication norfloxacin (Date et al., 2020). CQDPAs (Carbon quantum dot polyamines), a highly cationic form of Carbon quantum dot, are used to treat bacterial keratitis and infections due to their potent antibacterial properties (Jian et al., 2017). When compared to negatively charged CQDs, both uncharged and positively charged CQDs demonstrated superior bactericidal action synthesised from ethylene glycol and various amine sources (Verma et al., 2020). CQDs are synthesized from graphene-based materials (GQD) that exhibit characteristics of graphene and can be utilized for biomedical applications. Graphene-based CQDs have a lot of carboxylic groups, which enable them to be functionalized with active biomolecules (Kortel et al., 2020). This makes them useful for developing therapies and delivering drugs (Figure 11). The effectiveness of employing graphene-based CQDs as a low-cytotoxicity inhibitor for the aggregation of Aß peptides as they adhere to the hydrophobic centre of the peptides was demonstrated by Liu et al., 2015. Functionalized GQDs have demonstrated significant effectiveness in recognizing cancer receptors, transporting chemotherapeutic agents—like doxorubicin (DOX) or cisplatin—selectively to the cell nucleus while advancing the cytotoxicity, obstructing the agents’ unintentional transportation into normal cell tissues, and prohibiting drug resistance (Deng et al., 2018). Graphene-based CQDS widely used in photodynamic treatment (PDT) has shown significant therapeutic benefit (Choi et al., 2014). The molecules may be put into the system with the aid of the CQDs, opening the way to the prospect of drug administration with bioimaging capabilities. The functionalized CQDs’ biocompatibility remains a crucial concern for their future use in living cells, tissues, and animals (Tian et al., 2020). This could potentially be a disadvantage for the clinical testing of these CQDs in therapeutic applications in some cases. The disadvantages also include the synthesis of CQDs using different methods such as the need for costly ingredients, severe reaction conditions, and extended reaction times (Section 2).

### 6.2 Gene therapy

Gene therapy s the technique of modifying a person’s genes to treat or cure an illness (Pham et al., 2023). Gene therapy is regarded as a long-term and potentially curable therapeutic method for a variety of disorders (Dunbar et al., 2018). In this technique, the vector with excellent efficiency of gene transfection can transfer genetic components within the cells. According to reports from the past few years, CQDs can be employed as gene nanocarriers that can be tracked via imaging (Ghosh et al., 2019a; Han and Na, 2019; Pham et al., 2023). Therefore, among various nanoparticles, CQDs take considerable attention in gene therapy as a non-viral vector. Various research has revealed that desirable CQDs/polymeric nanostructures can be a suitable competitor for gene delivery in specific areas like tumours (Molaei, 2019). A hybrid nano-gene vector-based CQDs (CQD-PEI), produced utilizing glycerol along with the branched PEI25k through microwave-aided pyrolysis, has the superior capability to gene transfer because of the outer positive charged polymer layer on the CQDs (Liu et al., 2012). CQDs were employed as a gene carrier for chondrogenesis from fibroblasts (Molaei, 2019). The development of multifunctional nano-carriers has demonstrated potential for use in diagnostics and gene therapy. CQD complexed with PEI and unlabeled plasmid DNA presented real-time monitoring for gene delivery (Kim et al., 2013). While plasmid DNA and polymeric vectors can be labelled with organic dyes and inorganic QDs, research has demonstrated health issues and instability. When compared to PEI alone, CQDs capped with PEI matrix allowed plasmid DNA for a greater transfection into COS-7 and HepG2 cells (Zhu et al., 2022). The hydrogel demonstrated intriguing simultaneous imaging capabilities with several excitation wavelengths, suggesting potential applications for near-infrared emissions imaging *in vivo*.

Kisor et al. developed CQD-PAMAM conjugates (CDPs) by synthesizing CQDs using peels of sweet lemon as a renewable source and conjugating them with several generations of polyamidoamine (PAMAM) dendrimers (Ghosh et al., 2019b). Between the conjugates of CDP, CDP3 is a potential gene transfer vector for TNBC (triple negative breast cancer) gene therapy because of its better gene complexation along with protection abilities, low level of toxicity, compatibility of blood, and enhanced gene transfection effectiveness, resulting in an intriguing theranostic technique for future therapy (Ghosh et al., 2019b). Furthermore, CDP3 demonstrated extremely specific Cu (II) ion detection, which could assist in determining the metastatic stage of TNBC, which has a larger concentration of Cu (II) ions. To function as an effective pseudo-homogeneous gene transfer vehicle, Ehsan et al. constructed CQDs from chitosan which were functionalized with arginine (Rezaei and Hashemi, 2021). Due to robust photoluminescence properties in both solid and solution states under physiological environments and excellent cellular intake in the human embryonic kidney −293T (HEK-293T) cells without any toxicity, the CQDs decorated with poly-l-lysine (PLL), namely, CQD/PLL core-shell NPs, show significant potential in the application of gene delivery (Hasanzadeh et al., 2021). CQDs, made from sodium alginate, were successfully employed as an effective non-viral gene carrier (Zhou et al., 2016). These CQDs displayed transfection effectiveness that was comparable to lipofectamine, but they also had the added benefits of cell imaging, extremely minimal cytotoxicity, and good biocompatibility. It was also found that multifunctional nanoparticles of polyethyleneimine-based carbon quantum dots (PCD) have the potential to transmit genes within cells. Both sodium alginate-based CQD and PCD can bioimaging probes discussed earlier (Zhou et al., 2016; Han and Na, 2019).

### 6.3 Combatting COVID-19

COVID-19, a pulmonary viral disease causing severe pulmonary distress with fever, has led to massive global deaths recently (Zhou and Ye, 2021). Scientists are working on rapid diagnosis and therapy when immune cells produce mediators (i.e., interleukins, α-defensins) that induce inflammation (Zhou and Ye, 2021; Soltani-Zangbar et al., 2022). CQDs are employed to diagnose and treat the disease (Xue et al., 2022). The herbs are more effective than medical plants as the precursor of CQDs to fight COVID-19 (Łoczechin et al., 2019; Naik et al., 2022). As an example, curcumin-derived CQDs were effective against enterovirus (EV-71) and garlic-derived CQDs were later found to diminish inflammatory cytokines while reducing coronavirus attachment and penetration (Lin et al., 2019; Kalkal et al., 2021). CQDs have been synthesized by hydrothermal treatment of ethylenediamine and citric acid as precursors of carbon and post-modification with boronic acid ligands, which exhibited concentration-dependent coronaviral inactivation (Łoczechin et al., 2019). The nanoporous membranes made of carbon dots and poly- (vinylidene fluoride) for self-sterilized, recyclable facemasks to combat the virus have been reported (Singh et al., 2021). The composite films have a hydrophobic surface, compact nanopore network, and efficient filtering of particles larger than 100 nm. CQDs have had a significant role in combatting COVID-19.

Systems based on CQDs may find use in the future in antiviral and antibacterial applications. Targeted delivery of medicinal medications loaded on CQDs, biological dye, and biological nanotransporters will become more and more significant. Enhanced photocatalytic antibacterial activity of covalent organic frameworks (COFs) is reported for CQDs as an electron extractant, which encompasses a great possibility for collaboration among material scientists and biochemical researchers for advanced biological and bio-environmental applications (Das et al., 2022b; 2023; Liang et al., 2022).

## 7 Conclusion

This review provides insight into the various synthetic methods of CQDs, their structure and properties, and the applications of CQDs in bioimaging and biomedicine. The article focuses on recent advancements, future possibilities, and various applications of CQDs and CQDs-based composites, hetero-atom-doped modifications in the field of bioimaging and biomedicines.

The CQDs were found in 2004 during the purification of single-walled carbon nanotubes (SWCNTs) and sanitizing them. Since that time, there have been a lot of techniques reported for the synthesis of CQDs. In general, CQDs can be synthesized using either of two primary methods: top-down or bottom-up. Top-down approaches use physical or chemical mechanisms to split up bigger carbon structures into smaller ones (Wang et al., 2014). Because of their low preparation costs and various advantageous qualities, such as their solubility in aqueous medium, chemical inertness, biocompatibility, and non-toxicity, CQDs have since been used in a wide variety of applications. Right electron doping and right surface passivation on CQDs are utilised in drug delivery due to their biocompatible properties and fluorescent properties. CQDs can be used as carriers for drug delivery, tracking continuous fluorescence emission. We emphasized new applications of CQDs, focusing on the precursors, properties, and synthesis of CQDs following their uses in bioimaging and biomedicine. A variety of biomedical applications have come to rely on CQDs and carbon-based composite materials because of their distinct physiochemical, immune-quiescent, biocompatible, and other characteristics. The powerful light-absorbing ability of CQDs allows the targeted destruction of tumour cells and microorganisms in photothermal therapy, photodynamic therapy, and PTT/PDT combo treatments (Juzenas et al., 2008; Choi et al., 2014). This review’s primary contribution is the suggested framework for the future incorporates ascertaining diverse sources yielding CQDs with adaptable biomedical uses, comprehending the mechanism of action and eventual secretion through renal process from the body.

We have put a special emphasis on highlighting nanocomposites comprising CQDs and composites, featuring efficient uses for bioimaging and biomedicines. The fluorescence off/on the mode of CQD/polymer composites may be applied to detect bioanalytics, hence bringing information from the laboratory scale to commercial and industrial domains (Li W. et al., 2017; Ma et al., 2018; Adam et al., 2022). Preclinical research indicates promise for medication delivery, on-chip labs, cancer therapy, and non-invasive diagnostics. CQDs enable real-time monitoring of the subcellular and tissues, which makes it a quick and easy way to assess patients’ health and an area of great focus in research. Investigations into antivirals based on CQDs are still in their early stages. Currently, targeting and utilizing CQDs to prevent certain viruses is difficult. Research on CQDs-based single-atom nanomedicine aims to clarify the exact mechanism for bio applications and enhance complicated selectivity and loading efficiency. CQDs in polymer matrices (Carbonized Polymer Dot or CPD) and CQDs synthesized from graphene-based materials (GQD) are utilized for various bioimaging and biomedical applications (Liu et al., 2015; Pathak et al., 2023). This helps in the imaging process incorporating various wavelengths, red and NIR wavelengths, easing the drug delivery process, and gene therapy.

More research is needed on CQDs, which offer new applications in bioimaging and biomedicines. Although the synthesis methodologies and potential applications are greatly explored, tuning the sizes of CQDs for desired fluorescence properties and functionalization on CQDs in some cases in a simple, environmentally friendly method is challenging. Future research would focus on raising the QY of CQDs and developing clearly defined geometrically, compositionally, and structurally CQDs. Many unknown features in CQDs might open their extensive use in bioimaging and biomedicines to combat a range of threats in the future.
